# Learning from natural variation across the proteomes of single cells

**DOI:** 10.1371/journal.pbio.3001512

**Published:** 2022-01-05

**Authors:** Nikolai Slavov

**Affiliations:** 1 Department of Bioengineering, Northeastern University, Boston, Massachusetts, United States of America; 2 Barnett Institute, Northeastern University, Boston, Massachusetts, United States of America

## Abstract

Biological functions arise from protein interactions, which are reflected in the natural variation of proteome configurations across individual cells. This Perspective article argues that emerging single-cell proteomics methods may decode this variation and empower inference of biological mechanisms with minimal assumptions.

## Teaser

Biological functions arise from protein interactions, which are reflected in the natural variation of proteome configurations across individual cells. Emerging single-cell proteomics methods may decode this variation and empower inference of biological mechanisms with minimal assumptions.

We normally think of experiments as procedures carefully designed by scientists and engineers. However, some of the best experiments arise from natural processes. For example, evolution and gene segregation during reproduction can be seen as natural experiments that have revealed much about protein functions and about genetic associations with diseases [1]. A salient advantage of such natural experiments is that they provide empirical evidence that is inaccessible to human designed experiments because of ethical and technical limitations.

Single-cell proteomics will open a window to a vast data trove of natural experiments: the proteome configurations that reflect molecular interactions within and between our cells. Indeed, signals received by cells in our bodies are transmitted via networks of proteins, and the relationships among these proteins reflect the underlying regulatory process. The transmission often involves protein cleavages, additions of chemical groups (e.g., a phosphate group), and binding interactions among different protein partners. These molecular events shape the covariation among the involved proteins, and thus the protein configurations of single cells may report on biological functions and regulatory networks [2]. Therefore, the patterns of proteome configuration across single cells may reveal biological functions and their regulation.

**Fig 1:**
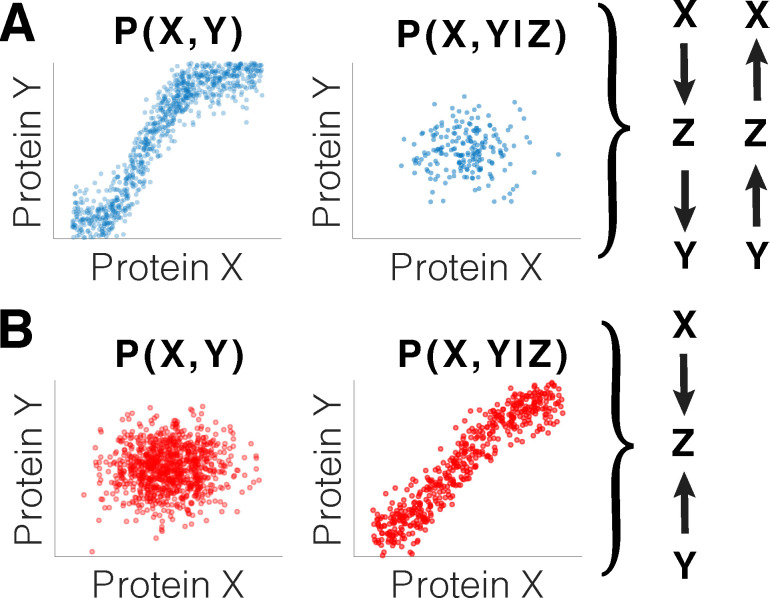
Inference of direct regulatory interactions with minimal assumptions. **(A)** The observed joint distributions of proteins X and Y across many single cells are consistent with 2 models in which protein Z is a confounder. **(B)** The joint distributions of proteins X and Y across many single cells are consistent with a “collider” model, in which X and Y collide at Z, inducing dependence conditional on Z. The arrows indicate directions of causality (positive or negative regulation), and in the collider model one of the arrows corresponds to positive and the other to a negative regulatory effect. Such inference of direct regulatory interactions requires no specific assumptions, but it does require accurate measurements across many single cells.

One such example is the possibility of inferring cell type–and cell state–specific protein complexes from covariation of protein levels across single cells. Covariation among the subunits of protein complexes is well established from analyzing bulk samples composed of mixed cellular states. Such covariation was among the first demonstrated results from Single-Cell ProtEomics by Mass Spectrometry (SCoPE-MS) [3]. Covariation is observed not only among the subunits of protein complexes but also among functionally related proteins that do not interact directly, and thus covariation alone is not a reliable indicator of protein interaction. Rather, it can be combined with additional measurements and informative features to infer direct regulatory interactions [4]. Compared to bulk measurements, single-cell proteomics offers 3 major advantages for exploring protein complexes. First, it allows inferring protein relationships specific to each of the cell types making up a complex tissue. Second, it can observe the full dynamic range of protein variation across cells, which is decreased by the averaging of bulk measurement. This full range should support stronger and more reliable correlations, although this possibility has not yet been demonstrated. Third, single-cell proteomics affords many data points (across single cells) to support robust estimates of covariation and may even support more general estimates from the joint distributions of protein abundances (Fig 1). Such estimates may allow modeling complex relationships without assuming the functional form of models and thus can reduce assumptions.

Another example of learning from single-cell variation is the inference of transcriptional regulatory interactions. These are among the most studied regulatory interactions over the last 2 decades because of the wealth of transcriptomic measurements [5]. Nonetheless, the joint analysis of proteins and transcripts across single cells can reveal regulatory interactions that cannot be identified only from single-cell RNA measurements [6,7]. Furthermore, joint analysis of protein and mRNA levels allows identifying proteins whose abundances are not well predicted by the corresponding RNA levels, such as the tumor suppressor p53 [7]. This type of inference must account for measurement noise, which demands new methods for modeling noise in single-cell proteogenomic analysis.

## Distinguishing between direct and indirect regulation

Analysis of single-cell protein variation may also enable a long elusive goal: the quantitative characterization of the direct protein interactions that weave the signal transduction networks in our cells. While genomics identifies many causal genetic associations, these associations are indirect [1]. Such indirect associations are mediated by many unobserved molecules and can be explained by very many different models. This multiplicity of models limits the utility of indirect causal associations [1].

By contrast, single-cell measurements of proteins may enable inference of direct regulatory interactions with minimal assumptions, as shown in Fig 1. Analysis of joint distributions of protein abundances can directly reveal regulatory interactions without depending on model assumptions. Specifically, conditioning the joint distribution of proteins X and Y and a confounder protein Z may reveal that while X and Y are correlated, they do not directly regulate each other (Fig 1A). Alternatively, the joint distribution of A and B may not be correlated unless conditioned on protein Z, which suggests a different regulatory model (Fig 1B). Importantly, this analysis does not require assuming specific types of models or functional dependencies. Rather, it requires quantitatively accurate protein measurements across many single cells. This requirement is becoming feasible due the technological advances discussed below.

## Technological requirements and frontiers

Learning from the patterns of single-cell protein variation requires accurate single-cell protein measurements. Indeed, ideas described here have previously surfaced in the literature [8], but their implementation has remained limited by the type and accuracy of the available high-throughput single-cell measurements. This is because the data analysis outlined above, such as conditioning joint distributions on confounders, is much less tolerant to measurement noise than the currently popular single-cell analysis methods, such as cell type clustering and dimensionality reduction.

Therefore, realizing these possibilities requires that we advance the technology. First, we must make the state-of-the-art methods widely accessible via detailed protocols, computational resources, and community standards [9]. Second, we must continue to increase the accuracy of the measurements by (i) increasing the sampling efficiency of protein molecules and thus reducing counting errors; (ii) reducing the potential for measurement interferences; and (iii) using control and reference samples that may allow reducing the impact of experimental artifact, as in the case of ratiometric measurements. This second requirement will take advantage of exciting opportunities for innovation that can increase the depth and accuracy of proteome profiling by intelligent data acquisition and parallel analysis of both peptides and single cells [10].

In addition to increasing the accessibility and accuracy, single-cell proteomic technologies will develop toward measuring protein dynamics, activities, and localization. For example, protein synthesis and degradation dynamics may be encoded into the proteomes of single cells by pulsing at different time points amino acids labeled with heavy isotopes. These encoded dynamics can be decoded at the end of the experiment by MS measurements of the abundances of proteins labeled with each isotopic composition. Protein localization may be measured by physically isolating cellular organelles, while posttranslational modifications (PTMs) may be analyzed by using PTM-enriched isobaric carriers [2,9]. Such analysis requires future technological advances, which will be powered by major opportunities for innovation [10].
